# The Rush Monument Committee

**Published:** 1888-05

**Authors:** 


The Rush Monument Committee of the American Medical Association will report progress at the next meeting of the association, at Cincinnati, on the 8th instant. We trust that that report will indicate that the medical profession of America is willing to honor the memory of one of the most illustrious members of the profession that America has produced. Other countries have not failed to perpetuate the memory of their illustrious physicians in this manner, but the omission, in this respect, on the part of America, that has been liberal in honoring others of her distinguished citizens, is the more conspicuous. The claims of Dr. Rush on all classes of citizens because of his great services, and their varied character, make it fitting that one who participated so prominently in the cause of our national independence, and who was an ornament to the medical profession, should be one of the first to be thus honored by those upon whom the honor of his life-record is reflected.

